# Editorial: Clinical evidence for and advances in translational research on the classic formulas of traditional Chinese medicine

**DOI:** 10.3389/fphar.2024.1392930

**Published:** 2024-04-15

**Authors:** Pengqian Wang, William Chi-shing Cho, Dewei Ye, Yuqing Zhang, Xingjiang Xiong

**Affiliations:** ^1^ Institute of Chinese Materia Medica, China Academy of Chinese Medical Sciences, Beijing, China; ^2^ Department of Clinical Oncology, Queen Elizabeth Hospital, Hong Kong, Hong Kong SAR, China; ^3^ Key Laboratory of Glucolipid Metabolic Diseases of the Ministry of Education, Guangdong Pharmaceutical University, Guangzhou, China; ^4^ Department of Clinical Epidemiology and Biostatistics, McMaster University, Hamilton, ON, Canada; ^5^ Department of Cardiology, Guang’anmen Hospital, China Academy of Chinese Medical Sciences, Beijing, China

**Keywords:** traditional Chinese medicine, classic formulas, Chinese herbal medicine, translational research, evidence-based medicine

## 1 Introduction

Traditional Chinese medicine (TCM) is one of the ancient healing practices with a history of more than 2,500 years and unique theories that has been making great contributions to human healthcare. It includes Chinese herbal medicine, food therapy, acupuncture, moxibustion, massage (*tuina*), and physical exercises, among others (Tang et al., 2008), and is becoming increasingly popular in other Asian and Western countries. The classic formulas of TCM have clear verifiable scientific literature on traditional usage and are generally recognized to be derived from ancient medical books, such as Treatise on Febrile Diseases (伤寒论, *Shang Han Lun*) and Synopsis of the Golden Chamber (金匮要略, *Jin Gui Yao Lue*). TCM is also popular in Japan and Korea, so the classic formulas of TCM are also widely used in traditional Japanese Kampo medicine and traditional Korean medicine. TCM possesses the characteristics of fixed components (generally greater than or equal to two herbs) and clear curative effects with fewer adverse effects in clinical treatment. The classic formulas of TCM have made substantial contributions to the wellbeing of mankind, including viral diseases, cardiovascular and cerebrovascular diseases, digestive diseases, endocrine diseases, and tumors.

## 2 Clinical evidence for the classic formulas of TCM

Currently, rigorous “totality of evidence” of the classic formulas of TCM that includes chemical standardization, biological assays, experimental studies, and clinical trials is receiving increasing attention from modern medicine. Several paradigms in the clinical and translational research efforts on the classic formulas of TCM, such as *Tianma Gouteng* decoction for hypertension (Zhang et al., 2020), *Qili qiangxin* capsule (*Shenfu* decoction) for chronic heart failure (Li et al., 2013), *Tian qi jiang tang* capsule (*Yuye* decoction) for impaired glucose tolerance (Lian et al., 2014), *Gegenqinlian* decoction for T2DM (Xu et al., 2015), *Hemp seed* pill for functional constipation (Zhong et al., 2018), *Tong xie yao fang*-based Chinese herbal medicine for irritable bowel syndrome (Bensoussan et al., 1998), *Maxingshigan* decoction-*Yinqiaosan* for H1N1 influenza virus (Wang et al., 2011), *Maxingshigan* decoction for COVID-19 (Hu et al., 2021), PHY906 (*Huangqin* decoction) for chemotherapy-induced gastrointestinal toxicity (Lam et al., 2010), and Shenfu injection for patients with return of spontaneous circulation after in-hospital cardiac arrest (Zhang et al., 2017), highlight the tremendous progress and advances in this field (as shown in Table 1). Furthermore, large amounts of clinical evidence have provided some biological functions and potential mechanisms of the classic formulas of TCM. These suggest that the classic formulas of TCM could be considered effective complementary and alternative approaches in the future.

**TABLE 1:** Clinical evidence and advances in translational research in Classic Formula of TCM.

Classic formula of TCM	Disease	Components	TCM efficacy	Pharmacological mechanism	Author’s comments	TCM classification	Additional explanation	References
*Tianma Gouteng* granule	Masked hypertension	Rhizoma Gastrodiae (*Tianma*), Uncaria (*Gouteng*), Concha Haliotidis (*Shijueming*), Eucommia Ulmoides Oliv (*Duzhong*), Loranthus Parasiticus (*Sangjisheng*), Achyranthes Bidentata Blume (*Niuxi*), Leonurus Japonicus (*Yimucao*), Gardenia (*Zhizi*), Scutellaria Baicalensis (*Huangqin*), Poria Cocos (*Fuling*), and vine of Multiflower Knotweed (*Shouwuteng*).	Calming the liver, suppressing wind, clearing heat, promoting blood circulation, and tonifying the liver and kidney.	Inhibiting sympathetic activity and renin-angiotensin-aldosterone system, blocking L-type calcium channels, improving the vasomotor function, and reducing inflammatory reactions.	The randomized, placebo-controlled trial of 251 participants showed that *Tianma Gouteng* granule could mildly reduce daytime and 24-h ambulatory blood pressure in patients with masked hypertension.	Classic formula of TCM, and Chinese patent medicine	*Tianma Gouteng decoction* is a classic formula invented by TCM physician *Guangci Hu* in New Meaning of Treating Miscellaneous Diseases and Syndrome in Internal Medicine of Traditional Chinese Medicine (*Za Bing Zheng Zhi Xin Yi*) about 70 years ago.	Zhang, et al. (2020)
*Qili qiangxin* capsule	Chronic heart failure	Astragalus Membranaceus (*Huangqi*), Ginseng (*Renshen*), Radix Aconiti Carmichaeli (*Fuzi*), Salvia Miltiorrhiza (*Danshen*), Lepidium Seed (*Tinglizi*), Alisma (*Zexie*), Polygonatum Odoratum (*Yuzhu*), Cassia Twig (*Guizhi*), Safflower Carthamus (*Honghua*), Cortex Periplocae (*Xiangjiapi*), and Dried Tangerine Peel (*Chenpi*).	Invigorating *qi*, warming *yang*, promoting blood circulation, dredging collaterals, and inducing diuresis to alleviate edema.	Increasing myocardial contractility, cardiac output, and renal blood flow, reducing ventricular wall thickness and heart index, lowering levels of angiotensin II and aldosterone, and alleviating ventricular remodeling	A multicenter, randomized, double-blind, placebo-controlled study of 512 patients with chronic heart failure demonstrated that *Qili* qiangxin capsules can further reduce the levels of NT-proBNP, and improve the left ventricular ejection fraction (LVEF), 6-minute walking distance (6MWD) and quality of life, suggesting that it could be used as combination therapy for chronic heart failure.	Chinese patent medicine	*Qili qiangxin* capsule is originated from classic formula *Shenfu* decoction, which is invented by TCM physician *Ji Xue* in *Zheng Ti Lei Yao* about 500 years ago.	Li, et al. (2013)
*Tian qi jiang tang* capsule	Impaired glucose tolerance	Astragalus Membranaceus (*Huangqi*), Trichosanthes Root (*Tianhuafen*), Ligustrum Lucidum (*Nvzhenzi*,), Dendrobium (*Shihu*), Ginseng (*Renshen*,), Wolfberry Bark (*Digupi*), Coptis Rhizome (*Huanglian*), Cornus Officinalis (*Shanzhuyu*), Eclipta (*Mohanlian*), and Chinese Gall (*Wubeizi*).	Tonifying qi and yin, clearing away heat, and promoting salivation.	Regulating the metabolism of glucose and lipid, improving insulin sensitivity, alleviating inflammatory injury, and regulating oxidative stress.	A randomized, double-blind, placebo-controlled trial of 420 patients with impaired glucose tolerance demonstrated that treatment with *Tian qi jiang tang* capsule for 12 months significantly decreased the incidence of T2DM, and this herbal drug was safe to use.	Chinese patent medicine	*Tian qi jiang tang* capsule originated from classic formula *Yuye* decoction, which is invented by TCM physician *Xichun Zhang* in Records of Traditional Chinese and Western Medicine in Combination (*Yi Xue Zhong Zhong Can Xi Lu*) about 110 years ago.	Lian, et al. (2014)
*Gegen qin lian* decoction	Type 2 diabetes	Pueraria Lobata (*Gegen*), Scutellaria Baicalensis (*Huangqin*), Coptis Rhizome (*Huanglian*), and Liquoric Root (*Gancao*).	Clearing away heat and dampness.	Improving insulin resistance, inhibiting intestinal inflammatory response, promoting intestinal mucosal repair, antioxidant stress, protecting islet β cells, and improving glucose and lipid metabolism.	A randomized, double-blind, controlled trial of 187 patients with type 2 diabetes demonstrated that *Gegen qin lian* decoction significantly reduced hemoglobin A1c and fasting blood-glucose.	Classic formula of TCM	*Gegen qin lian* decoction is a classic formula invented by TCM physician *Zhongjing Zhang* in *Treatise on Febrile Diseases* (*Shang Han Lun*) in *Han* dynasty about 1800 years ago.	Xu, et al. (2015)
*Maziren* pill	Functional constipation	Hemp Seed (*Huomaren*), Rheum Officinale (*Dahuang*), Fructus Aurantii (*Zhishi*), Magnolia Officinalis (*Houpu*), Bitter Apricot Seed (*Kuxingren*), and White Peony (*Baishaoyao*).	Moistening the intestines, clearing away heat, promoting *qi*, and relaxing bowels.	Improving defecation function and peptic protease activity.	A randomized, double-blind, double-dummy, controlled trial of 291 patients with functional constipation identified that *Maziren* pill is well-tolerated and effective in increasing complete spontaneous bowel movement/week.	Classic formula of TCM, and Chinese patent medicine	*Maziren* pill is also a classic formula invented by TCM physician *Zhongjing Zhang* in *Treatise on Febrile Diseases* (*Shang Han Lun*).	Zhong, et al. (2018)
*Tong xie yao fang*-based Chinese herbal medicine	Irritable bowel syndrome	Saposhnikovia Divaricata (*Fangfeng*), Dried Tangerine Peel (*Chenpi*), White Peony (*Baishaoyao*), White Atractylodes Macrocephala (*Baizhu*), Codonopsis Pilosula (*Dangshen*), Agastache rugosus (*Huoxiang*), Coix Seed (*Yiyiren*), Radix Bupleuri (*Chaihu*), Oriental wormwood (*Yinchen*), Magnolia Officinalis (*Houpu*), Baked Ginger (*Paojiang*), Ash Bark (*Qinpi*), Poria Cocos (*Fuling*), Dahurian Angelica Root (*Baizhi*), Plantain Seed (*Cheqianzi*), Phellodendron Amurense (*Huangbai*), Liquoric Root (*Gancao*), Costus Root (*Muxiang*), Coptis Rhizome (*Huanglian*), and Chinese Magnoliavine Fruit (*Wuweizi*).	Tonifying spleen, smoothing liver, dispelling dampness, and stopping diarrhea.	Anti inflammation, regulating immune function, mood and liver lipid metabolism, and improving intestinal hypersensitivity.	A randomized, double-blind, placebo-controlled trial of 116 patients with irritable bowel syndrome demonstrated that Chinese herbal medicine is effective in the management of symptoms related to irritable bowel syndrome.	Classic formula of TCM	*Tong xie yao fang* is a classic formula invented by TCM physician *Danxi Zhu* in *Danxi*'s Law of the Heart (*Dan Xi Xin Fa*) in *Yuan* dynasty about 700 years ago.	Bensoussan, et al. (1998)
*Maxingshigan* decoction-*Yinqiaosan*	H1N1 Influenza	Ephedra (*Mahuang*), Bitter Apricot Seed (*Kuxingren*), Gypsum (*Shigao*), Liquoric Root (*Gancao*), Honeysuckle bud and flower (*Jinyinhua*), Forsythia suspensa (*Lianqiao*), Lophatherum Stem and Leaves (*Danzhuye*), Fineleaf Schizonepeta Herb (*Jingjie*), Great Burdock Achene (*Niubangzi*), Fermented Soybean (*Dandouchi*), Field Mint (*Bohe*), Root of the Balloon Flower (*Jiegeng*), and Reed Rhizome (*Lugen*).	Clearing away heat and toxic material, and relieving the exterior syndrome.	Inhibiting bacteria, antiviral, anti-inflammatory, anti-allergic, antipyretic, and analgesic.	A prospective, nonblinded, randomized, controlled trial of 410 patients with laboratory confirmed H1N1 influenza identified that *Maxingshigan* decoction-*Yinqiaosan* reduce time to fever resolution, which could be used as an alternative treatment of H1N1 influenza virus infection.	Classic formula of TCM	*Maxingshigan* decoction is a classic formula invented by TCM physician *Zhongjing Zhang* in *Treatise on Febrile Diseases* (*Shang Han Lun*). *Yinqiaosan* is also a classic formula invented by TCM physician *Jutong Wu* in Item Differentiation of Warm Febrile Diseases (*Wen Bing Tiao Bian*) in *Qing* dynasty about 200 years ago.	Wang, et al. (2011)
*Lianhuaqingwen* capsule	Coronavirus disease 2019	Forsythia suspensa (*Lianqiao*), Honeysuckle bud and flower (*Jinyinhua*), Ephedra (*Mahuang*), Polygala Tenuifolia (*Yuanzhi*), Hawthorn (*Shanzha*), Rheum Officinale (*Dahuang*), Liquoric Root (*Gancao*), Cyrtomium fortunei (*Guanzhong*), Rhodiola rosea (*Hongjingtian*), Houttuynia cordata (*Yuxingcao*), Bitter Apricot Seed (*Kuxingren*), Gypsum (*Shigao*) and Menthol.	Clearing plague and detoxifying, promoting lung, and purging heat.	Broad spectrum antiviral, antibacterial and anti-inflammatory, reducing fever, relieving cough, resolving phlegm, and regulating immune.	A multicenter, prospective, randomized controlled trial including 284 patients with Covid-19 identified that the recovery rate, median time to symptom recovery, time to recovery of fever, fatigue and coughing, the rate of improvement in chest computed tomographic manifestations and clinical cure were significantly improved when compared to usual treatment.	Chinese patent medicine	*Lianhuaqingwen* capsule is a modified formula of *Maxingshigan* decoction, which is a classic formula recorded in *Treatise on Febrile Diseases* (*Shang Han Lun*).	Hu, et al. (2021)
*Huangqin* decoction	Chemotherapy-induced gastrointestinal toxicity	Scutellaria Baicalensis (*Huangqin*), White Peony (*Baishaoyao*), Liquoric Root (*Gancao*), and Jujube (*Dazao*).	Clearing away heat, and reliving diarrhea and pain.	Regulating immunity, inhibiting bacteria, anti-inflammatory, and anti-cancer.	*Huangqin* decoction can counteract the toxicity of irinotecan and reduce chemotherapy-induced gastrointestinal toxicity, especially diarrhea.	Classic formula of TCM	*Huangqin* decoction is a four herb formula recorded in *Treatise on Febrile Diseases* (*Shang Han Lun*). PHY906 (*Huangqin* decoction) has passed the U.S. FDA’s investigation new drug application and will enter Phase II human clinical trials.	Lam, et al. (2010)
*Shenfu* injection	Return of spontaneous circulation after in-hospital cardiac arrest.	Red ginseng (*Hongshen*) and Radix Aconiti Carmichaeli (*Fuzi*).	Revive the yang for resuscitation, tonifying qi, and preventing exhaustion.	Improving heart failure, anti myocardial ischemia-reperfusion injury, anti arrhythmias, anti shock, improving myocardial energy metabolism and microcirculation, protecting endothelial cells, improving hemodynamics, and regulating blood pressure.	A prospective, randomized, controlled clinical study of 1,022 patients demonstrated that *Shenfu* injection in combination with conventional postresuscitation care bundle treatment is effective in improving clinical outcomes in patients with return of spontaneous circulation after in-hospital cardiac arrest.	Chinese patent medicine	*Shenfu* injection is originated from classic formula *Shenfu* decoction which is recorded in *Zheng Ti Lei Yao* about 500 years ago.	Zhang, et al. (2017)

## 3 Classic formulas of TCM-oriented new drug development model

Owing to challenges in modern drug research and development such as improving investments and preventing the decline of drug approval, scientists have focused their attention on natural herbs and the classic formulas of TCM to create promising drugs and drug candidates. Derivatives from natural products have been widely used as cornerstones in Western medicine, such as aspirin, digitalis, and paclitaxel. Recently, natural herbs and the classic formulas of TCM-oriented new drug development model have achieved significant breakthroughs, including artemisinin and dihydroartemisinin for malaria (Tu, 2011), arsenic trioxide for acute promyelocytic leukemia (Soignet et al., 1998), tripterygium glycosides for active rheumatoid arthritis (Lv et al., 2015), red yeast rice for hyperlipidemia (Zhao et al., 2004), and berberine for hyperlipidemia and diabetes (Kong et al., 2004; Zhang et al., 2008); these can help avoid blind screening while reducing the cost and time required for drug development.

## 4 Advances in translational research on the classic formulas of TCM

Given the difficulty of retrieving original literature in Chinese as well as the poor methodological quality of the original clinical trials, such as high risk of bias, unclear reporting of outcome measures, lack of preregistration in an international database, lack of high-level clinical recommendation evidence, and insufficient data reports on the toxicology, pharmacological effects, and adverse effects, several classic formulas of TCM have been regarded as a “mystery” to modern science. Accordingly, more clinical and experimental data are warranted to verify their application values.

A total of 43 papers were received under this Research Topic, out of which eight papers were accepted for publication. The original research articles and reviews in this Research Topic cover a wide range of medical conditions, including essential hypertension, contrast-induced nephropathy after percutaneous coronary intervention, hepatocellular carcinoma, lung cancer, doxorubicin-induced cardiotoxicity, DNCB-induced atopic-dermatitis-like skin lesions, and heat stroke.

Two papers elaborate on the clinical applications of the classic formulas of TCM for cardiovascular diseases. Lin et al. provide clinical evidence on the Qiangli Dingxuan tablet, which is widely used in traditional Chinese patent medicine for the treatment of essential hypertension. Fu et al. evaluated the clinical improvement of contrast-induced nephropathy after percutaneous coronary intervention with the compound Danshen dripping pills, which could be used to relieve angina pectoris.

Three papers clarify the clinical efficacies and mechanisms of the classic formulas of TCM for tumor-based diseases. Luo et al. tested the protective effects of the Jianpi Huayu decoction for hepatocellular carcinoma. Shahid et al. investigated the effectiveness and mechanisms of the medicinal mushroom *Ganoderma lucidum* for lung tumorigenesis induced by the carcinogens in tobacco smoke. Wang et al. addressed the effects of the active ingredients in *Salvia miltiorrhiza* on doxorubicin-induced cardiotoxicity.

Additionally, Zhao et al. examined the therapeutic effects of the Fangji Dihuang formulation, a classic formula of TCM first described by Zhongjing Zhang (150–219 A.D.), for DNCB-induced atopic-dermatitis-like skin lesions. The remaining two articles focus on research into heat stroke and the quality markers of the classic formulas of TCM.

## 5 Conclusion

In recent times, steadily increasing amounts of evidence have shown that the classic formulas of TCM have important clinical value in the treatment of various conditions, including chronic and infectious diseases. Findings under this Research Topic can significantly promote comprehension of the therapeutic efficacies and potential protective mechanisms of the classic formulas of TCM. Thus, this Research Topic encompasses exploration of new clinical evidence and novel approaches in the application of the classic formulas of TCM to the treatment of various diseases, paving the evidence-based path for the future.
